# Game theory applications in host-microbe interactions toward disease manifestation: *Mycobacterium tuberculosis* infection as an example

**DOI:** 10.22038/IJBMS.2021.55471.12410

**Published:** 2021-10

**Authors:** Hiva Sharebiani, Sara Hajimiri, Shadi Abbasnia, Saman Soleimanpour, Amir Mohamad Hashem Asnaashari, Narges Valizadeh, Mohammad Derakhshan, Rezvan Pilpa, Arezoo Firouzeh, Kiarash Ghazvini, Saeed Amel Jamehdar, Seyed Abdolrahim Rezaee

**Affiliations:** 1 Immunology Research Center, Inflammation and Inflammatory Diseases Division, Mashhad University of Medical Sciences, Mashhad, Iran; 2 Antimicrobial Resistance Research Center, Bu-Ali Research Institute, Mashhad University of Medical Sciences, Mashhad, Iran; 3 Department of Microbiology and Virology, School of Medicine, Mashhad University of Medical Sciences, Mashhad, Iran; 4 Lung Disease Research Center, Mashhad University of Medical Science, Mashhad, Iran

**Keywords:** Game theory, Mycobacterium tuberculosis, Ag85, ESAT-6, Th1, Tuberculosis

## Abstract

**Objective(s):**

Game theory describes the interactions between two players and the pay-off from winning, losing, or compromising. In the present study, *Mycobacterium tuberculosis* (*Mtb*)–host interactions were used as an example for the application of game theory to describe and predict the different outcomes of *Mtb*-infection and introducing target molecules for use in protection or therapy.

**Materials and Methods:**

The gene expression for eight main markers (CCR1, CCR2, IDO, Tbet, TGFβ, iNOS, MMP3, MMP9) of host response and three *Mtb* virulence factors (Ag85B, CFP-10, ESAT-6) were assessed in broncho-alveolar lavage of TB^+^ and TB^-^ patients.

**Results:**

The players’ strategies in the “Nash equilibrium”, showed that Ag85B is the main virulence factor for *Mtb* in active phase, and also the most immunogenic factor, if the host can respond by high expression of T-bet and iNOS toward a Th1 response. In this situation, *Mtb* can express high levels of ESAT-6 and CFP10 and change the game to the latency, in which host responses by medium expression of T-bet and iNOS and medium level of TGF-β and IDO. Consistently, the IDO expression was 134-times higher in TB^+^s than the TB^-^s,and the T-bet expression,~200-times higher in the TB^-^s than the TB^+^s. Furthermore, *Mtb*-Ag85B had a strong positive association with CCR2, T-bet and iNOS, but had a negative correlation with IDO.

**Conclusion:**

Ag85B and maybe ESAT6 (without its suppressive C-terminal) should be considered for making subunit vaccines. And, preventing IDO formation in dendritic cells might be a novel target for immunotherapy of tuberculosis, to reduce the pressure of immune-suppression on Th1 responses.

## Introduction

When two organisms, individuals, parties, teams, or even countries interact, the outcome varies greatly, depending on the intention of each party involved (1). Attempts to quantify these interactions can be assessed, using game theory models. Game theory is a mathematical framework, describing the outcome (pay-off) resulting from specific interactions (game) between two individuals (players) (2, 3). In a biological context, game theory can describe interactions between a host and its parasite through epigenetic strategies, and the resulting pay-off from healthy, infected, or disease onset. The conflict is very complex, as both organisms are responsible for resisting and helping the associated species to survive (1-3). 

Bacteria and their host under stress may carry out the sophisticated principles of game theory, in order to decide whether to compromise or invade for elimination of the danger (4). Therefore, it is especially important for players to think about each other’s strategic choices and react accordingly(4). Interactions between *Mycobacterium tuberculosis* (*Mtb)* and human (players) are often included in the *Mtb’s* strategies to invade host responses, to replicate and persist within the host, and on the other hand, the host attempts to induce appropriate responses to eliminate the infection (5). Particularly, in the case of *Mtb* infection, these interactions are in a strict completion, as the microbe has adapted to replicate within the macrophages, which are specialized cells for killing microbes and the host must activate the infected cell to clear *Mtb* (5, 6). 

The nature of *Mtb* and the genetic characteristics of the host seem to be important in the development, progress, and severity of infection. Even though numerous association studies have been performed to identify genetic factors responsible for variations in TB susceptibility, to date, these candidate genes have not been found (7, 8). Therefore, *Mtb* elimination and dormant or active *Mtb* infections in a given population seem to be associated with epigenetic processes. With a better understanding of the connections between bacterial infectious diseases and epigenetic events, opportunities will arise for therapeutic solutions, particularly, as epigenetic processes can be reverted. This opens a new pathway for future research in the ﬁeld of microbial pathogenesis (9, 10). 

After infectious droplets of *Mtb* are inhaled, innate and adaptive effector mechanisms for intracellular infection or appropriate cell-mediated immunity (CMI) are able to eliminate the infection. The main effector player in this situation is the macrophages that are strongly activated by IFN-γ, and the habitats for *Mtb* transform into activated macrophages to eradicate the infection (11). 

In an immunological equilibrium or latent TB, the main immune response against *Mtb* is CMI, which can only control bacterial replication and consequent dissemination. A solid granuloma develops around the bacteria, composed of mononuclear immunity cells, such as macrophages in different maturation stages and T cells of different phenotypes (12). 

In reactivation or active TB infection, the weakening of the immune system or inappropriate immune responses causes the granuloma to become caseous and later to liquefy, and as a result, *Mtb* starts to replicate. Then, they leave their host cells and spread to other areas of the lung, other organs, and the environment (13), which is called active TB. 

In this study, the main elements in each phase of host immune responses and *Mtb* virulence factors were introduced in the game theory framework to show the application of this theory in host-microbe interactions. Briefly, the main strategies to adapt to its habitat, *Mtb*, as a parasitic organism, is to produce several virulence molecules, such as the 6-kD early secreted antigenic target ESAT-6 (EsxA), the 10-kD culture filtrate protein CFP-10 (EsxB), PPEs, and Ag85B, which actively intervene in both innate and adaptive immune responses of the host (14, 15). 

Chemokines (CCLs) and chemokine receptors (CCRs) are the first factors, in response to a danger signal involved in cell migration and immune polarization (such as Th1, Th2, and Th17). The polarization of these different types of responses depends on APC functions, which can activate specific transcription factors like T-bet, GATA-3, ROR-γt, and FoxP3 toward polarization (16, 17). For example, IDO activates in APCs, catalyzes tryptophan, and thus, can induce a Treg response to produce immune-modulatory TGF-β, or APC- producing IL-12 can induce a Th1 response to secret IFN-γ (18, 19). The last stage is activation of macrophages to produce oxygen-dependent, highly toxic factors for killing *Mtb*, proteases, inducing apoptosis or autophagy, or producing matrix metalloproteinases (MMPs), which may be involved in inflammation or tissue damages. (20-22). 

In tuberculosis infection, *Mtb* and the host are two players in a game with a conflict of interest, in which interaction of their strategies determines the outcome (23). Among all possible strategies, appropriate responses of each player were selected by Nash equilibrium calculation as rational and optimal responses, happening in our assessments, and other possible strategies were excluded due to complexity. The interest of *Mtb* is to enhance its payoff by replicating as much as possible, and then by being transferred out of the host to find a new subject. In contrast, the host attempts to decrease or eliminate the infection with minimal harm (23).

In the present study, *Mtb*-host interactions were used as an example for the application of the game theory to describe and predict the different outcomes of *Mtb*-infection and introducing target molecules for use in protection or therapy. Therefore, some main epigenetics factors were assessed and introduced to the game theory framework to describe the three outcomes of *Mtb-*host interactions, including host overcomes the infection (protection), equality (latency), or *Mtb* dissemination (active TB). 

## Materials and Methods

***Study population***

The study population was 30 patients, including 14 TB patients and 16 non-tuberculosis pulmonary patients with positive Mantoux test results who were referred to the Department of Internal Medicine, Ghaem Hospital, Mashhad University of Medical Sciences (MUMS), Mashhad, Iran. In the TB+ group, the smear and culture of sputum were negative, but IS6110-PCR on the BAL sample was positive. For each patient, a clinical examination and a checklist were completed by a pulmonologist. The study was approved by the Bio medical Ethics Committee of MUMS, (No: 941165, 930690, 930635, and 930634) and informed consent was obtained from each subject. In addition, all experiments were performed in accordance with relevant guidelines and regulations. In particular, written informed consent was obtained from all participants and all HIPAA identifiers were anonymized.

***Sample collection***

A total of 50 ml pulmonary lavage was taken from each subject by a pulmonologist. After centrifugation at 7000 RPM, the pellet was stored in the Tri-pure (Roche Co., Germany) at -70 ^°^C, until further RNA extraction. 

***RNA extraction and cDNA synthesis***

Total RNA was extracted from Tripure-treated samples (Roch Diagnostics Roche, Mannheim, Germany), according to the manufacturer’s instructions, and then reverse transcribed to cDNA with random hexamer primers, using the RevertAid™ H kit (Fermentas, Germany).

All primers/probes for gene expression analysis were designed, using sequence data available in the NCBI databases (NCBI, NIH, USA) by Beacon Designer software (PREMIER Biosoft International, Palo Alto, CA, USA). The specificity of the primers was checked by BLAST analysis (NCBI, NIH, USA). Primers and probes were synthesized by the BIONEER Corporation (South Korea), and their specificity was then confirmed by endpoint PCR and sequencing of the products by Applied Biosystems (SEQLAB, Germany). Table 1 shows the sequences of primers and probes for the main virulence antigens of *Mtb* and the immunological factors in the main steps of the anti-responses *Mtb*. 

***Relative real-time PCR***

Quantitative TaqMan real-time PCR was performed with a Q6000 machine (Qiagen, Hilden, Germany), using TaqMan premix (Takara Corporation, Japan) as previously described (24). Six standards were prepared using 10-fold serial dilution of a concentrated sample of the genes of interest and a reference gene [beta-2 microglobulin (β2M)]. The normalized value of the expression for each gene was calculated as the ratio of the number of relative copies of the mRNA of interest to the number of relative mRNA copies of the reference gene, indicated as the expression index.

***Statistical analysis***

The Statistical Package for Social Sciences (SPSS) version 13 (IBM SPSS Inc., Baltimore, MD, USA) was used for statistical analyses. Normality of the data was checked before data analysis using the Kolmogorov-Smirnov test. For non-parametric analysis, the Mann-Whitney U test and Kruskal-Wallis test were used to compare gene expression between the two groups. The Spearman correlation coefficient was used to show statistical dependence or correlation between variables. Data were presented as mean±SEM. Results were considered statistically significant if *P*≤0.05.

***Game theory mathematical modeling of the host and Mtb interaction***

**Figure 1:**
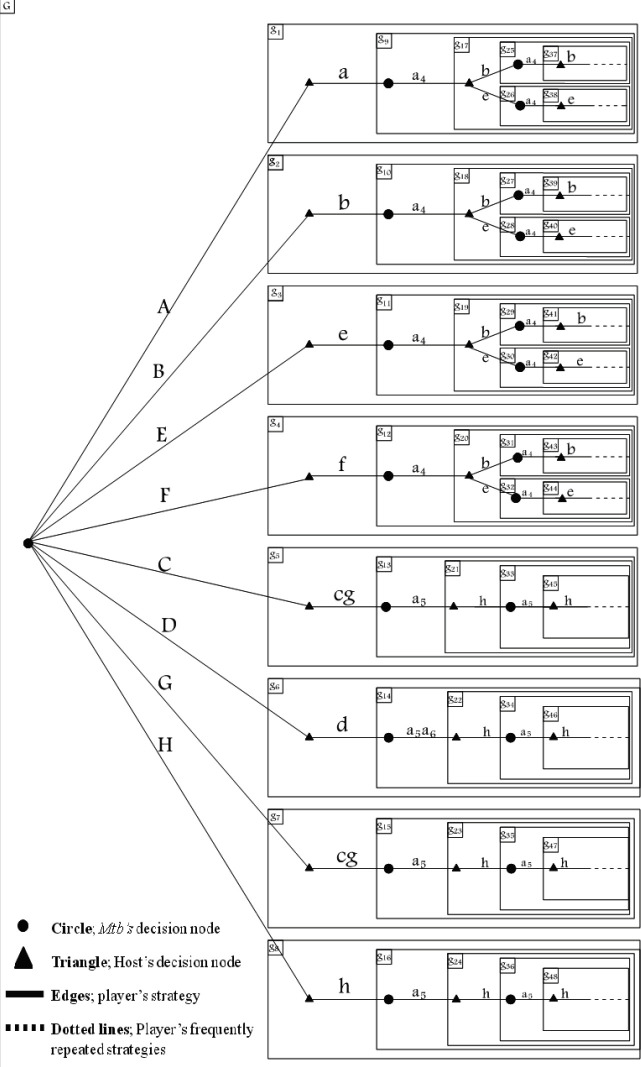
Circle; *Mtb’s* decision node. Triangle; Host’s decision node. Edges; Player’s strategy. …….. Dotted lines; Player’s frequently repeated strategies. G; Original game. g_n_; Subgame (shown by rectangular nesting). a_1_-a_6_; *Mtb*’s actions. A-H; *Mtb*’s strategies (final outcomes). a-h; Host’s strategies

The game theory was applied to the TB positive group, in which the main players in the onset of tuberculosis are the host and *Mtb*. Therefore, their interactions can be modeled in two strategic (Tables 2 and 3) and extensive forms (Figure 1) through backward induction. In all parts of the method, a dynamic game was assessed from the final (outcome) to the starting stages (initial interactions in infection). To design this game, the first step was to determine two types of interactions: sequential and simultaneous. Therefore, a stage game was considered, in which the players simultaneously performed a static subgame with complete information during each stage while present within a dynamic game and sequentially responding to their opponents (25-27).

***Quantification and design of the pay-off matrix***

The expression of main genes including host CCR1, CCR2, IDO, T-bet, iNOS, TGF-β, MMP3, and 9 plus *Mtb *Ag85B, CFP-10, and ESAT6 was considered as input data. Gene expression of selected genes was categorized (low/ high/ medium) by discretization by frequency operator and the pay-off matrix was designed based on conditional probability using the naive Bayes classifier (NBC) by Rapid Miner V5.3 software. 

***Designing the extensive form of the game based on subgame Perfect Nash equilibrium and Pareto efficiency***

The extensive form (or game tree) is a graphical representation of a sequential game. The game tree consists of nodes (or vertices), which are points at which players can take action. They are connected by edges, which represent the actions that may be taken at the node. An initial (or root) node represents *Mtb*’s strategies (S_A_-S_H_) which begin the game due to entrance and invasion of bacteria and perform as a trigger for starting an interaction with the host (game). Thus, pathways which are defined as every set of edges from the first node in the tree eventually arrive at the repetition stages called A-H based on this origination (*Mtb*’s strategies (S_A_-S_H_)). Pathways A-H consist of strategies that constitute a Nash equilibrium in every subgame of the original game, and this equilibrium is called the subgame perfect Nash equilibrium (SPE) (25). As explained earlier, the edges eventually arrive in repetition stages (shown as a dotted line in Figure 1) instead of a terminal node, because this is an infinitely repeated game, and the pay-off at the end of the game is calculated as a total pay-off and cannot be shown in the extensive form. In infinitely repeated games, the per-period interest rate (r) that results from game repetition is important, and players change the stage pay-off to the 

present value by a discount factor defined as $\delta =\frac{1}{1+r}$

 . 

In order to calculate the total pay-off of an infinitely repeated game, a function was first defined as the utility of strategy (u_i_(s)) in each stage game (t) for every period (n) as follows:

$U_{i}=U_{i}(S_{t}^{n})$ (1)

Then the player’s present value pay-off (V_i_) was calculated, and the total pay-off was defined as the sum of the present values in each stage of the game for any time period calculated as:



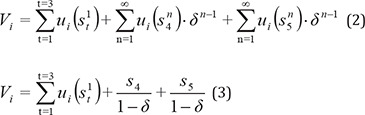



In infinitely repeated games, Patience means the increase of futute pay-off in the present time; on the other hand, with an increase in repetitions, r goes to zero. Then, δ goes to one, and future pay-offs resulting from a game repetition are preferred and more valuable than momentary pay-offs (28); this is called collusive pricing, which is a subgame Perfect Nash equilibrium outcome and leads to cooperation with Pareto dominant strategies (26).

***Parallel subgames’ Perfect Nash equilibrium and appropriate response efficiency***

An equilibrium in which the player’s strategy constitutes Nash equilibrium in every parallel subgame set of the original game is a parallel subgame Perfect Nash equilibrium (PSPE), which is equal to the appropriate response because it is determined by the maximization of the minimum pay-offs under consideration of all SPE strategies in a time period (26).

To determine the PSPE, the original game is separated into five parallel subgame sets, each of which refers to a stage. Nash equilibrium joint strategy pay-offs are placed on each set of a parallel subgame, and finally, the best strategy of every parallel subgame is selected with the Min-Max method as PSPE. When a PSPE is found in any set of parallel subgames, the defined PSPE efficiency index is calculated for each path (A-H) (26).

If “x” is the number of strategies that matched and “y” is the number of strategies that are unmatched with PSPE in unrepeated parallel subgames, then “n_0_” will be the number of unrepeated parallel subgames, so that n_0_=x+y (26).

If “n_r_” is the number of repeated parallel subgames that passed after the number of repetition times “t” and n_r_ = x’+y’, then “x’” is the number of strategies matched with the PSPE in repeated parallel subgames and “y’” is the number of strategies which are unmatched. 

For paths repeated from the first stage, x’/n_r_ represents the PSPE efficiency index in the repeated parallel subgames. In other paths, this index is represented by x/n_0_ calculated in unrepeated parallel subgames. Therefore, three types of strategies exist based on PSPE efficiency: PSPE dominant strategy (x>y or x’>y’), PSPE dominated strategy (x<y or x’<y’), and PSPE optimal strategy (x=y or x’=y’) (26). 

The meaning of dominant and dominated strategies here differs from Pareto dominant and dominated, as this classification of strategies is based on PSPE efficiency, while classification in the previous parts was based on Pareto efficiency (26).

The PSPE efficiency index for each subject of paths repeated from the first stage (L(t) function) and other paths (R(t) function) is placed in a range of values based on the number of repetitions:



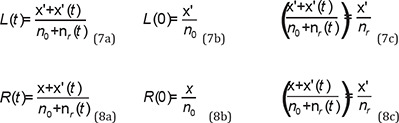



The PSPE efficiency index range for the path that earns “x” in unrepeated stages (n0) and “x’” in repeated stages (n_r_) is equal to [x/ n_0_, x’/n_r_), and the paths that are repeated from the first stage and earn “x’” have a constant value equal to x’/n_r_. 

***Cooperative and grim trigger strategies in the original game***

At the end of the modeling when all the last stages have been modeled, the game strategies are considered for the repeated stages and eight patient outcomes, and the earlier strategies that are placed in the repeated stages are cooperative strategies (29, 30). If one player contravenes his obligation, the first strategy that is not placed in the repeated stages is grim trigger strategy (28). The pay-off in a grim trigger strategy is less than that in cooperation strategies and could have different values of PSPE efficiency [x/ n_0_, x’/n_r_), while cooperative strategies are strategies with more pay-off that have a constant PSPE efficiency equal to x’/n_r_.

## Results

***Study population data***

The mean age of TB^+^ patients was 68.43±8.29 years (range=57 to 83 years), including 42.9% women. The mean age of TB^−^ controls was 69.56±7.46 years (range=57 to 83 years), and 56.3% were males and 46% were females. However, the differences in age and gender distribution were not statistically significant (Table 4). Table 5 shows the social and demographic characteristics of the subjects studied. Table 6 shows the comparison of the TB positive group with the TB negative group, in terms of clinical and para-clinical symptoms.

***mRNA levels of CCR1, CCR2, iNOS, T-bet, TGFβ, IDO-1, MMP3, and MMP9 in the host***

The CCR1 gene expression rates of BAL-PBMCs in TB^+ ^and TB^−^ patients were 1.1±0.23 and 0.34±0.09, respectively. There was a significant difference in the CCR1 gene expression between TB^+^ patients and TB^−^ controls *(P<0.01)*. Although, the average CCR2 gene expression in TB^+^ was around 3 times higher than in the TB^−^ subjects, no significant difference was observed in the 95% confidence level. The CCR2 gene expression rates of BAL-PBMCs in TB^+ ^and TB^− ^patients were 0.78±0.21 and 0.29±0.8, respectively *(P<0.01*) (Table 7).

The T-bet gene expression rates of BAL-PBMCs in TB^+^ and TB^− ^patients were 0.84±0.52 and 2.28±1.44, respectively; the difference was significant *(P<0.03)*. The average T-bet gene expression was around 1.53 times higher in TB^−^ patients than in the TB^+ ^subjects.

The mean mRNA expression rates of iNOS in the BAL-PBMCs from TB^+^ and TB^−^ patients were 0.64±0.29 and 1.72± 0.64, respectively. As shown in Figure 1, mRNA expression was around 2.68 times higher in healthy carriers than in the TB^+^ patients; however, the difference was not statistically significant (Table 7)*.*

The mean mRNA expression rates of TGFβ in the BAL-PBMCs of TB^+^ and TB^−^ patients were 0.2±0.07 and 0.139±0.03, respectively. As shown in Figure 1, mRNA expression was around 0.69 times higher in TB^+^ patients than in the healthy carriers* (P<0.85).* However, no significant differences were found in the TGFβ mRNA levels between TB^+^ and TB^−^controls (Table 7) (Figure 1).

The IDO-1 gene expression rates of BAL-PBMCs in TB^+ ^and TB^− ^patients were 0.67±0.28 and 0.034±0.01, respectively. The average IDO-1 gene expression in TB^+^ was around 19.70 times higher than in the TB subjects, and the difference was significant *(P<0.001) *(Table 7).

The mean MMP3 mRNA expression rates in the BAL-PBMCs of TB^+^ and TB^−^ patients were 0.22±0.09 and 0.64±0.23, respectively, and the difference was not significant (Figure 1) (Table 7). The MMP9 gene expression rates of BAL-PBMCs in TB^+^ and TB^−^ patients were 2.56±0.68 and 1.13±0.35, respectively. There was a significant difference in the MMP9 gene expression between TB^+^ patients and TB^−^ controls (*P<0.05*) (Table 7). The average MMP9 gene expression was around 2.26 times higher in TB^+^ patients than in the TB^−^ subjects. 

***Extensive form of game and three types of cooperation***

Figure 1 shows the extensive form of the game, in which the original game (G) is separated into subgames. Each path is an SPE of the original game. This is a Pareto dominant strategy, leading to cooperation and infinite repetition. There are three sets of strategies, each leading to specific repeated stages, consisting of a joint cooperative strategy. Every one of them shows one type of cooperation that equals latency strategies. The first set comprises the paths that begin with A, B, E, and F strategies and lead to “e” host’s strategy, in response to a high level of Ag85B, which is *Mtb’s* strategy (a4, e). The second set comprises the paths that begin with A, B, E, and F strategies and lead to the “b” host’s strategy, in response to a high level of Ag85B, which is also *Mtb’s* strategy (a4, b). The third set comprises the paths that begin with C, D, G, and H strategies, and lead to the “h” host’s strategy, in response to a high level of CFP-10, which is *Mtb’s* strategy (a5, h). The D path, however, led to “h” host’s strategy, in response to a high level of CFP-10 and ESAT-6, which is *Mtb’s* strategy (a5a6, h), before leading to “h” host’s strategy, in response to a high level of CFP-10, which is *Mtb’s* strategy (a5, h), just like C, G, and H paths.

***Evaluation of pathways by PSPE Efficiency As A Criterion For Appropriate Response***

PSPE was determined between parallel subgames in each stage, consisted of “h” strategy in t5, a4 (Ag85B (High)) *Mtb* strategy in t4, “h” host’s strategy against “a_5_a_6_” (high levels of CFP-10 and ESAT-6) *Mtb* strategy in stages t3 and t2, and finally in the paths that followed high levels of Ag85B (A, B, E, and F), “a” host’s strategy, and in other paths that followed high levels of CFP-10 and ESAT-6 (C, G, D, and H), “d” host’s strategy (Figure 1).

**Figure 2:**
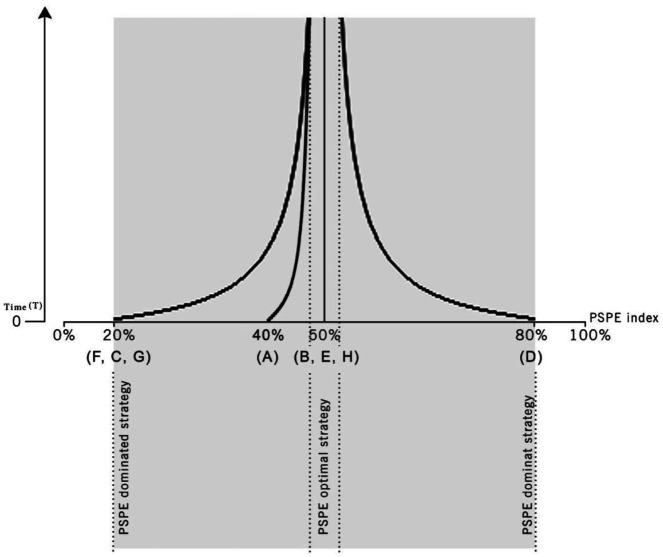
PSPE index during the time graph. Horizontal axis shows the PSPE index from zero until 100% and the vertical axis shows the number of repetition times from zero until infinity. The right curve is a PSPE dominant strategy (D path), left curves are PSPE dominated strategies (A, F, C, and G paths) and optimal strategy (B, E, and H paths) is a middle line that has a constant value during the repetition times equal to 50% and this is the vertical asymptote for two other curves. Increase in repeated times can lead to increases in the PSPE efficiency index and response appropriacy about inappropriate responses. This means that interaction between host and parasite will moderate by increasing the number of encounters of players together and players avoiding malicious strategies and moving towards cooperation strategies like increasing immune response inhibition by a medium level of TGF-β and IDO. Another argument is at least one of two players’ strategies which are in response to each other in repeated stages must be placed in PSPE to shape a cooperation strategy. In A, B, E, and F paths Mtb strategy is placed in PSPE, and in C, D, G, and H paths host strategy is placed in PSPE

Figure 2 shows the PSPE efficiency indices calculated for each path. This index had a 50% constant value in B, E, and H paths, classified into the PSPE optimal strategy (latent paths), 20% in the F, C, and G paths, and 40% in the A path, which is classified into the PSPE dominated strategy. The maximum value of this index was related to the D path (80%), which is classified into PSPE dominant strategy.

Moreover, this value can be calculated in each repetition for each path, as Figure 2 shows, in a time-course manner. Therefore, a long latency time could reduce the PSPE efficiency index to less than 80% in the D path, increase the PSPE efficiency index to higher than 20% in the F, C, and G paths, and increase it to more than 40% in the A path. Moreover, a long latency time is beneficial for the path that is classified into the PSPE dominated strategy, and a short latency time is beneficial for the path that is classified into the PSPE dominant strategy (Figure 2).

***Evaluation of the pathways by the level of MMPs expression***

To evaluate pulmonary tissue damage as a patient’s outcome of *Mtb*, the host interaction levels of MMP9 and MMP3 were determined and categorized into levels of low, medium, or high. Levels of MMP3 and MMP9 were determined in each path from A to H. The results showed that in the D path, the MMP9 level was classified into the low range; in the A path, it was classified into the low and medium ranges; the B and C paths were related to medium levels of MMP9, and G and F paths showed high levels of MMP9. Paths E and H had variable values. Paths B and F were classified into high levels of MMP3; D and C paths were classified into the medium range; the G path showed low levels of MMP3, and the H path was placed in the medium and low ranges. Paths E and A had variable values.

***Reactivation or grim trigger strategy***

In A, C, F, and G paths, the host contravened his obligation in the t1 stage and entered a grim trigger strategy, classified as a PSPE dominated strategy. The pay-off decreased and the PSPE efficiency index diminished from 50% to 20% for C, F, and G paths and from 50% to 40% for the A path. Therefore, reactivation was performed by inappropriate responses in C, F, and G, and finally led to a bad outcome (high levels of MMP9 in G and F and medium levels in C). In the A path, reactivation performed at a 40% efficiency. This is twice that of C, G, and F paths, even though it was placed on the PSPE dominated strategy. Yet, as a result, this path showed a better outcome with medium and low levels of MMP9.

In the D path, *Mtb* contravened his obligation (high levels of CFP-10 expression as a cooperative strategy) in the t2 stage, and entered a grim trigger strategy (high levels of ESAT-6 expression in addition to CFP-10), classifying as a PSPE dominant strategy. Because of this switching, pay-off decreased, and the PSPE efficiency index increased from 50% to 80%. Therefore, reactivation was performed by appropriate responses that led to better outcomes (low levels of MMP9).

According to the results, A, F, C, and G paths represented suitable strategies in latency that were reactive by the host and led to bad outcomes, while the D path represented a suitable strategy in latency that was reactive by *Mtb* and finally led to a good prognosis.

In B, E, and H paths, no player contravened from his obligation in any of t1 or t2 stages. Therefore, the players remained in their joint cooperative strategies with a constant pay-off and PSPE efficiency index (latent group).

**Table 1:** Primer and probe sequences & associated accession numbers

**Genes**	**Accession number**		**Primers**	**Probe **
**β2M**	^NM_004048.2^	F	^TTGTCTTTCAGCAAGGACTGG^	^TCACATGGTTCACACGGCAGGCAT^
		R	^CCACTTAACTATCTTGGGCTGTG^	
**CCR1**	^NM_001295.2 ^	F	^CCAGCATCTACCTCCTGAAC^	^ACCTGCTCTTCCTGTTCACGCTTC^
		R	^GGATCTTACACATGGCATCAC^	
**CCR2**	^NM_001123041.2^	F	^CGGCCTGAGTAACTGTGAAAG^	^ACTGGACCAAGCCACGCAGGTGACA^
		R	^CGAAGGCATAGATGATGGGATTG^	
**T-bet**	^NM_013351.1^	F	^GTCCAATGTGACCCAGATG^	^CCTCTGGCTCTCCGTCGTTCTCAACAC^
		R	^TGCGTGTTGGAAGCGTTG^	
**iNOS**	^NM_000625.4^	F	^GCTCAAATCTCGGCAGAATCTAC^	^TCCGACATCCAGCCGTGCCACCA^
		R	^GCCATCCTCACAGGAGAGTTC^	
**TGFβ**	^NM_000660.5^	F	^GCAAGTGGACATCAACGGGTT^	
		R	^CGCACGCAGCAGTTCTTCTC^	
**IDO**	^NG_028155.1^	F	^GCCAAGGTCATCCAACTACT^	^AAGCTGCTCGAGATTTCCACCAATAGA^
		R	^GCCTGCTTCACCACCTTCTGATG^	
**MMP3**	^NM_002422.4^	F	^CCCACTCTATCACTCACTCACAG^	^CCTGACTCGGTTCCGCCTGTCTCA^
		R	^CAAAGGACAAAGCAGGATCACAG^	
**MMP9**	NM_004994.2	F	^CAGCGAGAGACTCTACAC^	^CACCACGGACGGTCGCTCC^
		R	^GTCCCGGTCGTAGTTG^	
**Ag85B**	^NC_000962.3^	F	^CCTGCGGTTTATCTGCTCGA^	^AACACCCCGGCGTTCGAGTGGTACT^
		R	^TGTAGAAGCTGGACTGCCCG^	
**ESAT-6**	^NC_000962.3^	F	^GTCCATTCATTCCCTCCTTGAC^	^CCTGACCAAGCTCGCAGCGGC^
		R	^GCGTTGTTCAGCTCGGTAG^	
**CFP-10**	^NM_021803.3^	F	^GCCAATAAGCAGAAGCAGGAAC^	^CCTGCTGCTGCTCCTCGTCGGC^
		R	^GCCCATTTGCGAGGACAG^	

**Table 2:** Strategic form of game

**Group**	**Age (years)**		**P**
	Mean	SD	
**TB** ^+^	68.43	8.29	0.650
**TB** ^-^	69.56	7.46	
**Total**	69.03	7.74	

N; Players scape. *i*; player. A_i_; action profile of player *i*. a_n_ ; Action of *Mtb*. a’_n_; Action of host. S_i_; Strategy profile of player *i*. s; Strategy. U*i*; Pay-off profile of player *i*. G; Profile of subgames. g;Subgame. T; Profile of stages. t; Stage of game.

^†^a_1_= Ag85B (low), a_2_=CFP-10(low), a_3_=ESAT-6(low), a_4_=Ag85B (High), a_5_=CFP-10(High), a_6_=ESAT-6(High)

Ag85B (Low)< 0.082, Ag85B (High)> 0.082, CFP-10(Low)< 0.1, CFP-10(High)> 0.1, ESAT-6(Low)< 0.467 and ESAT-6(High)> 0.467.

^††^a’_1_=CCR1(low), a’_2_=CCR2(low), a’_3_=T-bet(low), a’_4_=iNOS(low), a’_5_=TGF-β(low), a’_6_=IDO(low), a’_7_=CCR1(Medium), a’_8_=CCR2(Medium), a’_9_=T-bet(Medium), a’_10_=iNOS(Medium), a’_11_=TGF-β(Medium), a’_12_=IDO(Medium), a’_13_=CCR1(High), a’_14_=CCR2(High), a’_15_=T-bet(High), a’_16_=iNOS(High), a’_17_=TGF-β(High), a’_18_=IDO(High)

CCR1(Low)< 0.51, CCR1 (Medium)= [0.51-1.12], CCR1(High)> 1.12, CCR2(Low)< 0.22, CCR2 (Medium)= [0.22-0.88], CCR2(High)> 0.88, T-bet(Low)< 0.003, T-bet(Medium)= [0.003-0.055], T-bet (High)> 0.055, iNOS(Low)< 0.003, iNOS (Medium)= [0.003-0.12], iNOS (High)> 0.12, TGF-β (low)< 0.068, TGF-β(Medium)= [0.068-0.155], TGF-β(High)> 0.155, IDO (Low)< 0.11, IDO(Medium)= [0.11-0.43] and IDO(High) > 0.43.

^‡^SA: Ag85B(Low), CFP-10(Low), ESAT-6(Low), SB: Ag85B(High), CFP-10(low), ESAT-6(Low),

SC: Ag85B(Low), CFP-10(High), ESAT-6(Low), SD: Ag85B(Low), CFP-10(Low), ESAT-6(High),

SE: Ag85B(High), CFP-10(High), ESAT-6(Low), SF: Ag85B(High), CFP-10(Low), ESAT-6(High),

SG: Ag85B(Low), CFP-10(High), ESAT-6(High), SH: Ag85B(High), CFP-10(High), ESAT-6(High),

^‡‡^S_a_: CCR1(High), CCR2 (Medium), T-bet (High), iNOS (High), TGF-β (Low), IDO (Low)

S_b_: CCR1(High), CCR2 (High), T-bet (High), iNOS (High), TGF-β (Medium), IDO (Medium)

S_cg_: CCR1(Medium), CCR2 (Medium), T-bet (Medium), iNOS (Medium), TGF-β (Low), IDO (Low)

S_d_: CCR1 (High), CCR2 (Medium), T-bet (Medium), iNOS (Medium), TGF-β (Low), IDO (Low)

S_e_: CCR1 (Medium), CCR2 (High), T-bet (High), iNOS (High), TGF-β (Medium), IDO (Medium)

S_f_: CCR1 (High), CCR2 (High), T-bet (High), iNOS (High), TGF-β (Low), IDO (Medium)

S_h_: CCR1 (Medium), CCR2 (High), T-bet (Medium), iNOS (Medium), TGF-β (Medium), IDO (Medium)

**Table 3:** Evaluation of action roles in host strategies

**The socio-demographic profile**			**TB** ^+^ ^N (%)^	**TB** ^-^ ^N (%)^	**TOTAL** ^N (%)^
**Sex/ gender**		Men	57.1	43.7	50
		Women	42.9	56.3	50
**Job**	**Men**	Employee	0	42.8	20
		Self-employed	75	57.2	66.6
		Retired	25	0	13.4
	**Women**	Housewife	42.8	64.2	50
		Employee	0	0	0

Evaluation of action roles in host strategies. Expression of each gene in every host strategy was specialized in all strategies that remained latent (B, E and H paths), levels of TGF-β and IDO placed in medium range and in other paths that reactivated, these genes show low expression. Therefore, levels of TGF-β and IDO were differentiator between latent and reactivated paths. In all host strategies in response to high levels of Ag85B Mtb strategy, T-bet and iNOS expressed in high level while these levels were medium in response to high levels of CFP-10 or CFP-10 and ESAT-6, therefore levels of T-bet and iNOS expression related with type of Mtb strategies. Levels of CCR1 and CCR2 related to increase and decrease of Mtb virulence gene expression. High level of CCR1 expression was related to expression of CFP-10 and ESAT-6 in low level and medium level of this chemokine receptor had reverse effect on expression of these virulence genes. When CCR2 expressed in high level, expression of Ag85B placed on high level too, against in medium levels of CCR2 such as C, G and D paths, expression of Ag85B placed on low level

Subgame perfect equilibrium extensive form of game shows backward moving from repeated strategies (latency), which was considered as the beginning of the game extended to the eight different *Mtb*’s strategies (A-H), by changing Ag85B, CFP-10, and ESAT-6 expression levels (reactivation or remaining latency). They are considered as final outcomes of host-*Mtb* interaction in each pathway. Host’s strategies “a”, “f”, and “cg” are grim trigger strategies and “a_5_a_6_” is another grim trigger strategy performing by Mtb, which is responded as strategy “d” by host. These strategies are reactivation strategies in favor of *Mtb* resulting in TB manifestation. Other *Mtb* and host strategies are cooperative strategies that are related to the latency phase

**Table 4:** Age homogeneity of groups

**Comparison of two groups in terms of clinical symptoms**									
**Clinical Symptoms**	**TB** ^-^				**TB** ^+^				**P**
	**YES**		**NO**		**YES**		**NO**		
	Frequencies	%	Frequencies	%	Frequencies	%	Frequencies	%	
**Cough**	15	93.75	1	6.25	14	100	0	0	0.533
**Dyspenia**	14	87.50	16	12.50	11	78.57	3	21.43	0.433
**Lymphadenopathy**	0	0	16	100	0	0	14	100	-
**Weight losing**	7	43.75	9	56.25	8	57.14	6	42.86	0.358
**Decreased appetite**	13	81.25	3	18.75	8	57.14	6	42.86	0.15
**Respiratory failure**	0	0	16	100	0	0	14	100	-
**Comparison of two groups in terms of para-clinical symptoms**									
**Clinical symptoms**	**TB** ^-^				**TB** ^+^				**P**
	**SD**		**Mean**		**SD**		**Mean**		
**Albumin**	0.58		3.98		0.08		4.44		0.02
**LYM**	8.77		36.24		11.37		26.44		0.01
**PMN**	8.36		57.22		12.39		69.48		0.004
**WBC**	2.21		7.6		2.4		6.8		0.30
**ESR**	11.78		35.64		14.60		28.87		0.1
**CRP**	0.51		0.5		0.12		0.5		1

**Table 5:** Social and demographic characteristics of the study subjects

**In human**				
**Genes**	**TB** ^+^ ^(N = 14)^	**TB** ^-^ ^(N = 16)^	**P**	
	X ± SEM	X ± SEM		
***CCR1***	1. 1±0.23	0.34± 0.09	0. 01	
***CCR2***	0.78± 0.21	0.29± 0.8	0. 1	
***T-bet***	0.84±0.52	2.28±1.44	0.03	
***INOS***	0.64±0.29	1.72± 0.64	0.24	
***TGF-β***	0.2± 0.07	0.139±0.03	0.85	
***IDO-1***	0.67 ± 0.28	0.034±0.01	0.001	
***MMP-3***	0.22±0.09	0.64±0.23	0.52	
***MMP-9***	2.56±0.68	1.13±0.35	0.05	
**In ** ***Mycobacterium tuberculosis***				
**Genes**	**N**	**Minimum**	**Maximum**	**X ± SEM**
***CFP-10***	13	0.003	0.452	0.11 ± 0.3
***Ag-85B***	14	0	13.79	1.16 ± 0.97
***Esat-6***	14	0.082	31.10	2.18 ± 8.19

**Table 6:** Comparison of TB positive group with TB negative group in terms of clinical and para-clinical symptoms

N= {*Mtb*, Host}
A*Mtb*= {a_1_, a_2_, a_3_, a_4_, a_5_, a_6_} ^†^
S_Mtb_= {s_A_, s_B_, s_C_, s_D_, s_E_, s_F, _s_G_, s_H_} ^‡^
A_Host_= {a'_1_, a'_2_, a'_3_, a'_4_, a'_5_, a'_6_, a'_7_, a'_8_, a'_9_, a'_10_, a'_11_, a'_12_, a'_13_, a'_14_, a'_15_, a'_16_, a'_17_, a'_18_} ^††^
S_Host_= {s_a_, s_b_, s_cg_, s_d_, s_e_, s_f_, s_h_} ^‡‡^
U*i*= {u_Mtb_, u_Host_}
G= {g_n_, g_n+8_, g_n+16_, g_(2n-1)+24_,g_(2n)+24_, g_(2n-1)+36_,g_(2n)+36_│n≤4}
G= {g_n_, g_n+8_, g_n+16_, g_(n+4)+24_, g_(n+4)+36_│4<n≤8}
T = T= {t_1_, t_2_, t_3_, ...∞│t_2_=t_2n_, t_3_=t_2n+1_}

**Table 7:** The gene expression data using real-time PCR

TGF-β and IDO (Medium)= latency	T-bet, iNOS (High)= Ag85B (high)		T-bet, iNOS (Medium)= CFP-10(High)	
	CCR1(High) CCR2(High) B MMP9(Medium) MMP3(High)	CCR1(Medium) CCR2(High) E MMP9(Variable) MMP3(Variable)	CCR1(Medium) CCR2(High) H MMP9(Variable) MMP3(Variable)	
TGF-β and IDO (Low)= Reactivation	CCR1(Medium) CCR1(High) CCR2(High) CCR2(Medium) F A MMP9(High) MMP9(Medium and low) MMP3(High) MMP3(Variable)		CCR2(Medium)=Decrease of Ag85B	
			CCR1(Medium) C G MMP9(Medium) MMP9(High) MMP3(Medium) MMP3(Low)	CCR1(High) D MMP9(Low) MMP3(Medium)

## Discussion

In this study, the status of host–microbe interactions (game) in the onset of TB (win or lose) was investigated in active and latent stages of infection. 

Therefore, to use game theory, we had to choose suitable variables, which are implicated in microbe and host strategy by evaluation of gene expression (epigenetics outcome) for each player (*Mtb* and host) in each subject (TB^+^), simultaneously. Therefore, the gene expression of the main virulence factor of *Mtb* (Ag85B, CFP-10, and ESAT-6) was considered as *Mtb*’s strategy, and the host’s main immunological molecules were assessed in four different steps of immune responses as host’s strategies: (i) leukocyte recruitment by chemokine receptors (CCR1 and CCR2), (ii) polarization of the response by transcription factor expressions (T-bet and TGF-β), (iii) cytokine and immunomodulatory factor production (IDO and iNOS), and (iv) release of effector molecules in tissue damage (MMP3 and MMP9). However, this condition can be changed depending on host (human) or microbe (*Mtb*) activities, under the influence of epigenetic strategies (31). In the present study, the first step of the game was induced by *Mtb* as a parasite for colonization in which the host responds by recruitment of leukocytes to the site of infection. Findings showed that both CCR2 and CCR1 expression were higher in TB^+^ patients than in Mantoux positive TB^−^ patients. Although CCR1 expression was statistically significant, CCR2 met significance only at level of 90% confidence interval (0.1). This form of expression is in favor of bacteria as Th2-like cells and monocyte macrophages recruit and exacerbate airway inflammation. 

The next step was consistent with these findings as the main difference between TB^+^ and TB^−^ patients was expression of T-bet, which was ~200 times more in the Mantoux positive TB^−^ patients than in the active TB patients. Furthermore, expression of IDO as an immune-suppressor was 134 times more in TB^+^ than in the TB^−^ patients. In such a situation, with a high level of IDO expression as an inducing factor for Treg polarization, a higher expression of TGF-β in TB^+^ patients is expected, while in reality, its expression was 4 times more, but did not meet the 95% CI (*P*=0.1). Taken together, Th1 recruitment (T-bet high expression) can protect the host against *Mtb *infection and Th2 like, Treg high responses and maybe the CCR2 expressing monocyte/macrophage have been in favor of lung inflammation and microbe dissemination in TB^+^ patients.

The expression profile for the TB^+^ patients was applied in a mathematical model to describe players’ behaviors in the game, and by the Pareto efficiency criteria to describe the three outcomes of *Mtb* infection, in terms of host-microbe interactions. In this model, an extensive form of the game was designed by SPE to exclude non-credible threats that a rational player would not actually carry out because it would not be in his interests (32). Therefore, none of the players selected low levels of defective factors or virulence molecules as a strategy, although these strategies were also not seen among the players, in the assessed situations. 

In this study, it could be assumed that all patients (TB^+^ and TB^−^) were in the latent phase, based on the mean age of the subjects and positive Monteux test results. At this stage of latency, the interactions of host and microbes reach a cooperation strategy or latency status, but some subjects experienced *Mtb *reactivation or grim trigger strategy. Some patients followed the strategies, in response to high levels of *Mtb* Ag85B expression (b and e strategies; Figure 1), and others designed different strategies in response to high levels of *Mtb* CFP-10 expression (h strategy; Figure 1).

 The best strategy for* Mtb* in latency is the high level of Ag85B expression that was determined to be a component of PSPE. This can be explained by the fact that Ag85B is a TAG synthase and might be a key player in both cell wall stability and biosynthesis of storage compounds for the survival of *Mtb,* in the dormant state (*Mtb* in the A, B, E, and F paths followed this strategy) (Figure 1).

Another strategy that *Mtb* may use in latency is high levels of CFP-10 expression. In this stage, all ESAT-6 molecules were in the form of CFP-10/ESAT-6 as a chaperone and were not able to express any effective function (C, D, G, and H paths followed this strategy). As a result, high levels of CFP-10 expression and the heterodimer form of CFP-10/ESAT-6 guarantee the compromise between the host and *Mtb*, due to (i) inhibition of phagosome rupture and cytosolic translocation of mycobacteria that prevent growth, proliferation, and dispersion of bacteria; (ii) ESAT-6 in complex with CFP-10 also interacts with beta-2 microglobulin (β2M), affecting the antigen presentation activities of macrophages and preventing the recognition of *Mtb* in dormancy; (iii) CFP-10 in the ESAT-6/CFP-10 complex may particularly contribute to neutrophil recruitment and activation. Thus, during *Mtb* infection, the high level of this virulence factor leads to inflammatory reactions, complicating the immuno-pathogenesis of TB (33); (iv) ESAT-6/CFP-10 complex enhances the production of NO and IL-12 released from M1 cells, following IFN-γ stimulation. The game theory results for this status showed that the presence of CFP-10 resulted in the production of medium level T-bet, inducing Th1 cells to produce IFN-γ, and also medium level of iNOS for NO production (7, 33). These two mechanisms keep the pathogen in a limited activity (latency); and (v) in such strategy, the high level of CCR2 expression may induce IFN-γR1 on the surfaces of the macrophages and create an appropriate innate immune response to help with *Mtb *elimination (16). 

In pathways C and G, when the host contravenes his obligation from “h” to “cg” strategy, leads to reactivation by a grim trigger strategy (cg), instead of a cooperative strategy (h). Furthermore, the production of IDO and TGF-β was decreased, in order to up-regulate the protective immune response to remove *Mtb*, also the probability of phagosome acidification increased, and CFP-10 and ESAT-6 were dissociated upon acidification. Consequently, ESAT-6 allowed interaction with the phagosomal membrane, resulting in phagosome rupture and cytosolic translocation of mycobacteria. Another altered part of the host strategy is the decrement of CCR2 expression, leading to the decreased recruitment of monocytes. This is a rational decision because ESAT-6 was shown to directly bind to Toll-like receptor 2 (TLR2), inhibiting TLR signaling in macrophages. Inhibition of macrophage response by ESAT-6 does not earn a significant pay-off (34). Therefore, the recruitment of monocytes will be decreased in the host, in order to prevent the pay-off of energy loss. This is a rational way to minimize *Mtb* pay-off by not allowing the bacteria to develop its own strategy.

In pathway D, the patient induces a pattern of gene expressions, including CCR1 (high), CCR2, T-bet, iNOS, TGF-β, and IDO (medium), in response to *Mtb’s* high expression of CFP-10 in the latent phase. In this commensal situation, *Mtb* decides to leave the cooperation and expresses ESAT-6 along CFP-10, leading to the increased probability of ESAT-6 production without CFP-10. Therefore, these ESAT-6 molecules can inhibit phagosome maturation and lead to phagosome rupture and cytosolic translocation of mycobacteria. With this strategy, bacteria can evade the toxic elements of the phagolysosome, and they can activate pro-inflammatory pathways such as IL-1, IL-8, IL-12, and IFN-β. Thus, this is an appropriate host immune response to low levels of TGF-β and IDO, high levels of CCR1, and medium levels of CCR2. That is a punishment strategy for *Mtb,* because (i) low levels of TGF-β and IDO lead to a decrease in immune system over-reaction by inhibiting harmful inflammation; (ii) change of CCR1 from the medium to the high level, consequently led to decreased CFP-10, as activation of this pathway recruits Th1 lymphocytes to the site of infection, which are the most powerful arm of the immune system to combat intracellular invaders. The radical change in CCR2 from a high to a medium level did not allow Th2 to come to the site of the infection (35). Consequently, the response promotes in favor of Th1, and the host can produce appropriate anti-*Mtb* responses. Thus, *Mtb* loses the game and has to decrease its production of the main virulence factor, Ag85B, due to the high level of immunogenicity. Finally, *Mtb* could just save a high level of ESAT-6, because the virulence is weak for dominance; it is a punishment state for *Mtb*. Moreover, low levels of MMP9 and medium levels of MMP3 in this path, show a low level of pulmonary tissue damage, in favor of a modulated proper immune response. 

Low levels of MMP9 lead to increased recruitment of neutrophils and an appropriate innate immune response, while medium levels of MMP3 can be related to the medium level of CCL2 (MCP-1), as a ligand of CCR2 and to medium levels of monocytes recruitment (36-39).

The game theory model demonstrated that the best outcome in path “D”, occurred for several reasons. The first one is related to the Pareto efficiency, like the other paths, but with only one difference, which is 80% of its strategies were matched with PSPE and appropriate protective responses. The rationale behind the reason that this strategy is better for the host is, unlike the other paths, in path D *Mtb* leaves the cooperation or latency by a grim trigger strategy, which is matched with PSPE. Firstly, *Mtb* is a parasite and the most common behavior for any parasite is to “save its host”, in favor of species survival. Reaching an equilibrium state with the host (latency) is the best strategy for *Mtb*, and perhaps for the host. 

If *Mtb* changes the strategy to a very severe infection, not only the host loses the game and dies due to the severity of the infection, but also the parasite loses its ecological niche, which is not in favor of the survival of the species. Therefore, adaptation of the host and the parasite by cooperation strategy for survival is rational as game theory showed by PSPE optimal paths B, E, and H at 50% efficiency. On the other hand, the parasite must have an evasion strategy not to lose the game. Thus, as the results of the current study showed, *Mtb* never changed strategy from high levels of CFP-10 to high levels of Ag85B. Although Ag85B is a powerful virulence factor for the bacteria, it is also an immune-dominant antigen for the host and induces a severe response to eliminate the bacteria. As a result, replacing high levels of ESAT-6 and CFP-10 with high levels of CFP-10 was the best decision, because ESAT-6 is a weaker virulence molecule than both Ag85B and CFP-10 and even an immune-modulator for host Th1 responses (40, 41).

The second response is the host’s response to high levels of ESAT-6 and CFP-10, differing from latency strategies, in terms of TGF-β and IDO levels. Furthermore, it differs from other reactivation strategies, in response to high levels of CFP-10, high levels of CCR1, and medium levels of CCR2 (41).

Among the paths (A, C, G, and F), in which the host contravenes his obligation and the new strategy is PSPE dominated, the path A reactivation strategy was more efficient and comparable with path D. In fact, A and D paths are analog, which are appropriate responses to high levels of Ag85B and CFP10-ESAT-6, respectively. 

In pathway A, the *Mtb* virulence factors in the lower levels and host strategies are a, b, and e, which can be frequently repeated in b and e. As can be concluded from these figures, *Mtb *first starts the colonization, and the host induces a higher expression of CCR1 and CCR2, T-bet, iNOS, IDO, and TGF-β expression rates are medium. In such a situation, *Mtb* tries to induce high expression of Ag85B (a4) frequently, until it reaches a host strategy (“a”), in which CCR1, T-bet, and iNOS remain at high levels, but CCR2 is at a medium level, and TGF-β and IDO are at a lower level. In these levels, the patients leave the cooperative strategy or latency, and *Mtb* can win the game with a higher expression of Ag85B, in which the players enter, a grim trigger strategy with 40% PSPE efficiency, which is 10% less than the cooperative strategy. Therefore, it should be noted that the host leaving a commensal interaction, results in a better situation for *Mtb* to escape from the host’s immune responses and enters reactivation status. It should be noted that the host’s strategies in these paths, only differ in the levels of T-bet and iNOS; however, TGF-β and IDO levels remain unchanged, which is in favor of *Mtb* reactivation. 

The lower levels of IDO and TGF-β can be in favor of the host, in clearing the infection. As previously mentioned, IL-10 and TGF-β are also important anti-inflammatory cytokines, modulating immune responses (42). Appropriate amounts of TGF-β and IL-10 (like medium level in latency strategies) in the *Mtb*-infected organs are vital, for balancing the Th1 protective response and preventing Th1 hypersensitivity (DTH) reactions, by suppressing the intensity of this response to bacteria. Thus, in *Mtb* infection, the importance of IL-10 and TGF-β may be double-edged (42, 43). High expression of TGF-β and IDO is a non-credible strategy that is excluded in this model since either dampening the host’s inflammatory response or limiting the Th1 appropriate response goes toward immuno-compromise, which may allow reactivation and dissemination of infection to an active TB or reactivation of a latent status. According to the current results, it can be expected that, in the presence of high levels of IDO expression, as an inducing factor for Treg polarization, TGF-β production will also increase significantly, but in gene expression data, this assumption did not meet this status (CI=90%, p=0.1). Of note, patients in this study had no immuno-compromising conditions (42).

Another change in the host response is related to the CCRs expression that leads to changes in cell recruitment of Th1, Th2, Treg, and Th17 for clearance, infection establishment, or granuloma formation (44). Besides physically sequestering mycobacteria, the formation of granuloma inhibits bacterial growth by subjecting mycobacteria to stressful conditions, such as starvation, reactive oxygen, nitrogen intermediates, and hypoxia. However, viable, apparently extracellular bacteria can persist in chronic granulomas for many years (44, 45). In a suitable status like an immune-compromised host, *Mtb* can overcome the pressure of the host’s immune system and be reactivated. As this model shows, there are direct associations between the strength of virulence factor, Ag85B, CFP-10, and ESAT-6, and the host’s immune response, such as CCR-1, T-bet, Th1 polarization, IFN-γ production, and iNOS, for macrophage activation.

The potentiation of the innate immune response in this model was determined by IFN-γ and iNOS, leading to NO production, and was responsible for the potentiation of the phagocytes for elimination of pathogens. 

For the last stage of the effector phase, MMPs assessment is the outcome of the responses in clearance or pulmonary tissue damage, which can be discussed in the context of the pathways C, G, and D. It should be noted that MMPs are also able to modify chemokine and cytokine activity, resulting in modified inflammatory cell recruitment (22, 46).

The immune response in both paths C and G had the same high levels of CFP-10, but the expression of MMPs differs. In path C, the medium level of MMPs contributes to the remaining *Mtb *infection*. *In path G, in the presence of the low level of MMP3, CCL2 degradation decreased, providing more CCL2 ligands for the medium expression of CCR2 (same levels as in paths C and G than path C). Therefore, monocytes are recruited more to the inflamed site of infection in the G path than to path C and do not allow Th2 to come. Consequently, the response is in favor of Th1, and the host can produce a strong appropriate reaction. On the other hand, a high level of MMP9 causes damage to pulmonary tissue, and by increasing degradation of IL-8, it decreases neutrophils’ recruitment at the infection site. In this strategy, the host does not have an appropriate innate immune response. Finally, in path G, high levels of ESAT-6-CFP-10 induce this outcome. Low levels of MMP9 and medium expression of MMP3 contribute to an appropriate innate response, in producing a moderate Th1 response and monocyte recruitment. 

The study has some limitations, firstly, such samples who are eligible for TB^-^ and TB^+^ are rare to have pulmonoscopy, which is an aggressive method. Secondly, life and its maintenance are some of the most complicated phenomena in the world, and science has not found a reliable methodology for the study of the complexity. Particularly, “the conflicts on survival in parasitism” in which behavior of two living players in the site of interactions must be studied, at the same time. It is nearly impossible to include all of the factors which are implicated in the site of microbe-host and micro-environment interactions in a three-dimensional system. Therefore, to introduce the game theory as a model for prediction of the outcome of such interactions, only main *Mtb* and host activities were included. 

## Conclusion

According to the “Nash equilibrium”; although Ag85B is the main virulence factor of *Mtb* for proliferation and winning the game, the most immunogenic factor arises when the host can respond by high expression of T-bet and iNOS and defeats the microbe. In such a situation of immune response, *Mtb* can express high expression of ESAT-6 and CFP10 and drives the game to a host strategy with a medium expression of T-bet and iNOS. In addition to these host factors, it is more likely that TGF-β and IDO are differentiating factors between latent and reactive phases, the medium expression levels of which can lead to latency and low levels to reactivation.

Accordingly, it can be assumed that effective vaccines, containing Ag85B and maybe ESAT6 (without suppressive C-terminal) should be considered in different attempts to protect the host from *Mtb* infection. Furthermore, with respect to host responses, application of the appropriate adjuvants and assessment of expression of T-bet and maybe CCR2 in PBMCs, in the presence of the multi-stage vaccine cocktail will be in favor of Th1-response. 

## Authors’ Contributions

RS and SH designed the study. SH, HS, AS, VN, PR, FA, and AH performed the experiments and analyzed the data. RS, SH, and SS wrote the paper. AH as a pulmonologist took the lavage. All authors have read and approved the final version of the manuscript.

## Funding

This research received no specific grant from any funding agency in the public, commercial, or not-for-profit sectors.

## Conflicts of Interest

The authors declare no conflicts of interest in this study. 

## References

[1] von Neumann J MO (1944). Theory of games and economic behaviour.

[2] Schmidt EFaKM (1999). A theory of fairness, competition, and cooperation. Quarterly J Economics.

[3] Lambert G, Vyawahare S, Austin RH (2014). Bacteria and game theory: The rise and fall of cooperation in spatially heterogeneous environments. Interface Focus.

[4] Tago D, Meyer DF (2016). Economic game theory to model the attenuation of virulence of an obligate intracellular bacterium. Front Cell Infect Microbiol.

[5] Ehrt S, Rhee K, Schnappinger D (2015). Mycobacterial genes essential for the pathogen’s survival in the host. Immunol Rev.

[6] Gengenbacher M, Kaufmann SHE (2012). Mycobacterium tuberculosis: Success through dormancy. FEMS Microbiol Rev.

[7] Peddireddy V, Doddam SN, Ahmed N (2017). Mycobacterial dormancy systems and host responses in tuberculosis. Front Immunol.

[8] Cheepsattayakorn A, Cheepsattayakorn R (2009). Human genetic influence on susceptibility of tuberculosis: From infection to disease. J Med Assoc Thai.

[9] Kathirvel M, Mahadevan S (2016). The role of epigenetics in tuberculosis infection. Epigenomics.

[10] Esterhuyse MM, Linhart HG, Kaufmann SH (2012). Can the battle against tuberculosis gain from epigenetic research?. Trends Microbiol.

[11] Khademi F, Derakhshan M, Yousefi-Avarvand A, Tafaghodi M, Soleimanpour S (2018). Multi-stage subunit vaccines against Mycobacterium tuberculosis: An alternative to the BCG vaccine or a BCG-prime boost?. Expert Rev Vaccines.

[12] Ahmad S (2011). Pathogenesis, immunology, and diagnosis of latent Mycobacterium tuberculosis infection. Clin Dev Immunol.

[13] Lenaerts A, Barry CE, Dartois V (2015). Heterogeneity in tuberculosis pathology, microenvironments and therapeutic responses. Immunol Rev.

[14] Tang XL, Zhou YX, Wu SM, Pan Q, Xia B, Zhang XL (2014). CFP10 and ESAT6 aptamers as effective Mycobacterial antigen diagnostic reagents. J Infect.

[15] Forrellad MA, Klepp LI, Gioffré A, Sabio y García J, Morbidoni HR, Santangelo MdlP (2013). Virulence factors of the Mycobacterium tuberculosis complex. Virulence.

[16] Domingo-Gonzalez R, Prince O, Cooper A, Khader S (2016). Cytokines and chemokines in Mycobacterium tuberculosis infection. Microbiol Spectr.

[17] da Silva MV, Massaro Junior VJ, Machado JR, Silva DAA, Castellano LR, Alexandre PBD (2015). Expression pattern of transcription factors and intracellular cytokines reveals that clinically cured tuberculosis is accompanied by an increase in mycobacterium-specific Th1, Th2, and Th17 Cells. Biomed Res Int.

[18] Blumenthal A, Nagalingam G, Huch JH, Walker L, Guillemin GJ, Smythe GA (2012). M. tuberculosis induces potent activation of IDO-1, but this is not essential for the immunological control of infection. PLoS One.

[19] Boer MC, Joosten SA, Ottenhoff THM (2015). Regulatory T-Cells at the Interface between human host and pathogens in infectious diseases and vaccination. Front Immunol.

[20] Rivera-Marrero CA, Schuyler W, Roser S, Ritzenthaler JD, Newburn SA, Roman J M (2002). tuberculosis induction of matrix metalloproteinase-9: The role of mannose and receptor-mediated mechanisms. Am J Physiol Lung Cell Mol Physiol.

[21] Lam A, Prabhu R, Gross CM, Riesenberg LA, Singh V, Aggarwal S (2017). Role of apoptosis and autophagy in tuberculosis. Am J Physiol Lung Cell Mol Physiol.

[22] Ong CW, Elkington PT, Friedland JS (2014). Tuberculosis, pulmonary cavitation, and matrix metalloproteinases. Am J Respir Crit Care Med.

[23] Eswarappa SM (2009). Location of pathogenic bacteria during persistent infections: Insights from an analysis using game theory. PLoS One.

[24] Kianmehr M, Rezaei A, Hosseini M, Khazdair MR, Rezaee R, Askari VR (2017). Immunomodulatory effect of characterized extract of Zataria multiflora on Th1, Th2 and Th17 in normal and Th2 polarization state. Food Chem Toxicol.

[25] Indrajit Raya SS (2013). Observable implications of Nash and subgame-perfect behavior in extensive games. J Math Econ.

[26] Nash J (1951). Non-cooperative games. Ann Math.

[27] Sorin S (1992). Chapter 4 Repeated games with complete information. Handbook of Game Theory with Economic Applications.

[28] Barton L (2009). Lipman RW. Switching costs in infinitely repeated games. GEB.

[29] Smith JM (1974). The theory of games and the evolution of animal conflicts. J Theor Biol.

[30] R Axelrod WH (1981). The evolution of cooperation. Science.

[31] Ip M, Zheng L, Leung ET, Lee N, Lui G, To KF (2015). Human epigenetic alterations in Mycobacterium tuberculosis infection: A novel platform to eavesdrop interactions between M. tuberculosis and host immunity. Hong Kong Med J.

[32] Heifetz A (2012). Game Theory interactive strategies in economics and management.

[33] Esmail H, Barry CE, Young DB, Wilkinson RJ (2014). The ongoing challenge of latent tuberculosis. Philos Trans R Soc Lond B Biol Sci.

[34] Pathak SK, Basu S, Basu KK, Banerjee A, Pathak S, Bhattacharyya A (2007). Direct extracellular interaction between the early secreted antigen ESAT-6 of Mycobacterium tuberculosis and TLR2 inhibits TLR signaling in macrophages. Nat Immunol.

[35] Hirsch CS, Rojas R, Wu M, Toossi Z (2016). Mycobacterium tuberculosis induces expansion of Foxp3 positive CD4 T-cells with a regulatory profile in tuberculin non-sensitized healthy subjects: Implications for effective immunization against TB. J Clin Cell Immunol.

[36] Rodriguez D, Morrison CJ, Overall CM (2010). Matrix metalloproteinases: What do they not do? New substrates and biological roles identified by murine models and proteomics. Biochim Biophys Acta.

[37] Salgame P (2011). MMPs in tuberculosis: Granuloma creators and tissue destroyers. J Clin Invest.

[38] Moores RC, Brilha S, Schutgens F, Elkington PT, Friedland JS (2017). Epigenetic regulation of matrix metalloproteinase-1 and -3 expression in Mycobacterium tuberculosis infection. Front Immunol.

[39] Shi C, Pamer EG (2011). Monocyte recruitment during infection and inflammation. Nat Rev Immunol.

[40] Karbalaei Zadeh Babaki M, Soleimanpour S, Rezaee SA (2017). Antigen 85 complex as a powerful Mycobacterium tuberculosis immunogene: Biology, immune-pathogenicity, applications in diagnosis, and vaccine design. Microb Pathog.

[41] Guo S, Xue R, Li Y, Wang SM, Ren L, Xu JJ (2012). The CFP10/ESAT6 complex of Mycobacterium tuberculosis may function as a regulator of macrophage cell death at different stages of tuberculosis infection. Med Hypotheses.

[42] Soleimanpour S, Farsiani H, Mosavat A, Ghazvini K, Eydgahi MR, Sankian M (2015). APC targeting enhances immunogenicity of a novel multistage Fc-fusion tuberculosis vaccine in mice. Appl Microbiol Biotechnol.

[43] Sia J, Rengarajan J (2019). Immunology of Mycobacterium tuberculosis i nfections. Microbiol Spectr.

[44] Flynn JL, Chan J, Lin PL (2011). Macrophages and control of granulomatous inflammation in tuberculosis. Mucosal Immunol.

[45] Shim D, Kim H, Shin SJ (2020). Mycobacterium tuberculosis infection-driven foamy macrophages and their implications in tuberculosis control as targets for host-directed therapy. Front Immunol.

[46] Muefong CN, Sutherland JS (2020). Neutrophils in tuberculosis-associated inflammation and lung pathology. Front Immunol.

